# Before allyship: A model of integrating awareness of a privileged social identity

**DOI:** 10.3389/fpsyg.2022.993610

**Published:** 2022-12-07

**Authors:** Jude Bergkamp, Lindsay Olson, Abi Martin

**Affiliations:** School of Applied Psychology, Counseling, and Family Therapy, Antioch University Seattle, Seattle, WA, United States

**Keywords:** social privilege, identity awareness, development, grounded theory, qualitative inquiry

## Abstract

**Introduction:**

Although the American Psychological Association encourages clinical psychologists to recognize and understand the experience of social privilege both within themselves and the individuals and communities they serve, there is a dearth of research in the field to guide this pursuit. According to the available literature, an essential barrier to social privilege integration is its implicit and covert nature that prevents consistent consciousness due to hegemonic forces.

**Methods:**

This study explored the process, from initial social privilege awareness to the moment of the study, through individual interviews. A social-constructivist, grounded theory approach was utilized as it was aligned with the understudied phenomena oriented around social justice.

**Results:**

The result is a developmental model of social privilege integration that explicates accumulated exposures to privilege, the resultant threat to and protection of personal identity, and the conducive factors that lead to reconciliation.

**Discussion:**

Implications of this theoretical model include the importance of a developmental perspective to cultivate an understanding of individual prejudice attitudes and discriminatory behaviors, as well as a roadmap toward equitable change. This model may be used by clinical psychologists across multiple settings in response to the most recent APA multicultural guidelines.

## Introduction

Academic spaces within clinical psychology have not kept up with America’s increased attention to social privilege. The past decade’s rise of social media, the influence of social-media users under age 40, and the racial reckoning of 2020 have intensified attention to the social use of terms like privilege, white privilege, and racial privilege. Attention to identity privileges—primarily racial—as historically invisible reinforcers of oppression has mostly come from social psychologists, sociologists, educators, counselors, and rarely from clinical psychologists (Lewis, 2004; Watt, 2007; Pinterits et al., 2009; Todd et al., 2011; McIntosh, 2012; Case et al., 2013; Goodman, 2015; Littleford and Jones, 2017; Johnson, 2018). This literature reveals a lack of clarity in how the term “social privilege” is defined and used as a broader construct that could unify anti-oppressive discourse and practice across academic and professional areas of the field. Empirical efforts to explain how individuals with social privilege make meaning of it are rarer still (Stoudt, 2009). The conspicuous lack within clinical psychology is perhaps due to the adoption of the medical model which relies on biological and behavioral rather than social and structural conceptualizations of individual identity and suffering.

The American Psychological Association (2017) recently published an update to the Multicultural Guidelines in which they recognize the effect of “historical and contemporary experiences with power, privilege, and oppression” (p. 4). These new guidelines represent the first time the APA as an organization has mentioned or endorsed the inclusion of privilege and power in research, education, and practice. The foundational work of scholars, such as McIntosh (1989), Tatum (1997), Helms (1984, 2017), Liu (2011), Spanierman and Smith (2017), Goodman et al. (2004), Goodman (2015), Case and Cole (2013), and Case (2012, 2017) suggest it is critical for psychologists to begin reflecting on their social privilege awareness to provide ethical and multiculturally competent treatment and services. The American Psychological Association (2017) further encourages not only a self-reflective process in alignment with these authors, but also a broader understanding of the forces of privilege and oppression that operate at large. Yet psychologists who in good faith seek to incorporate an understanding of power, privilege, and oppression into developmentally- and culturally-informed research, education, or practice are often faced with a dearth of empirical evidence on social privilege.

Although APA clearly suggests psychologists examine the social and historical bases of their personal attitudes and beliefs, and encourages a developmental approach to understanding these aspects of personal identity within themselves and how they affect clients, organizations, and institutions, it offers neither a clear invitation nor a roadmap for psychologists to engage in this obscure and isolating personal process. As clinical psychologists both in training and in practice, we set out to build a bridge from the aspirational goals of our guiding organization to our daily practice in clinical and educational settings. We hoped to learn more about the basic social process for anyone—not just psychologists—coming to terms with a privileged social identity so that we might apply our findings to our own daily lived experiences in relationship with others with privileged social identities.

A clear and universal definition of social privilege would be a helpful foundation for this bridge. Black and Stone (2005) provide an accessible definition of social privilege:

First, privilege is a special advantage; it is neither common nor universal. Second, it is granted, not earned or brought into being by one’s individual effort or talent. Third, privilege is a right or entitlement that is related to a preferred status or rank. Fourth, privilege is exercised for the benefit of the recipient and to the exclusion or detriment of others. Finally, a privileged status is often outside of the awareness of the person possessing it. (p. 244)

Social privilege is tied to the ranks of an individual’s positionality, also called social location, which “offers that all persons have a position in relation to others within a society” (Hearn, 2012, p. 42). An individual’s positionality is socialized by their overlapping social identities of race, sex, class, gender, ability, age, religion, sexual orientation, indigenous heritage, and nationality (Hays, 2016), and the meanings these identities have within society. These socialized positionalities contribute to an individual’s beliefs, attitudes, biases, and preferences. An individual might occupy a rank of social privilege in some of these identity domains, thus being afforded special rights in society, and a rank of social oppression in others, thus being denied special rights.

Terminology describing social location varies across time and discipline. Examples range from ascribed *status* and performative *role* (for a discussion of these terms and their limitations, see Van Ausdale and Feagin, 2001, p. 32–33) to racial *identities* in Sociology (see Omi and Winant, 1994, 2015). Alternately, psychology adds culturally determined *rank*—either *agent* or *target*—and interactional status (Nieto, 2010) while Education elaborates with *perspectivity* (Collins, 2003). For the purposes of this exploration, we will use *rank* primarily to refer to one’s social position in society and *agent* or *target* to refer, respectively, to a privileged or oppressed social position. In terms of intersectionality, individuals can hold multiple agent and target ranks within various social identities (Crenshaw, 2017). It is common to hold both agent and target ranks within an individual, and all of us experience some privileged agent ranks in terms of age, as we grow from childhood (target) into adulthood (agent) and elderhood (target again).

We consider that social privilege exists in corollary to oppression, whereas Black and Stone’s definition does not explicitly describe this important relationship. Goodman (2015) summarizes:

There is a relationship between the dynamics of being advantaged and disadvantages [sic]—some groups are advantaged *because* other groups are disadvantaged. People from privileged groups often benefit *at the expense of* people from oppressed groups…Oppression and privileged [sic] are two sides of the same coin. (p. 9, emphasis in original)

Oppression and privilege are inextricable, which creates some complications for understanding either as a stand-alone concept. However, it is cognitively—and often emotionally—easier to think of these concepts one at a time and then link them as understanding deepens. Many individuals tend to think of their positionality in terms of their oppressed ranks, even if their positionality also incorporates social identities that bestow privilege. This exemplifies the fifth aspect of definition of privilege of Black and Stone (2005) that individuals who possess privilege are often unaware of it. The most common metaphors used when discussing social privilege include the invisible knapsack (McIntosh, 2012), a fish in a river (Maxwell, 2004), and a tailwind (Kimmel, 2009). In other words, due to the nature of social privilege, we do not feel the weight of oppression, we take the quality of our environment for granted, and we neglect to acknowledge the forces that propel us.

A possible psychologically descriptive explanation for why privilege awareness is so elusive is dysconsciousness concept of King (1991). Although Goodman (2015) links privilege and oppression generally, King connected Black and Stone’s final condition of privilege to the cognitive and subsequent behavioral reinforcement of systems of oppression when she described dysconsciousness:

Dysconsciousness is an uncritical habit of mind (including perceptions, attitudes, assumptions, and beliefs) that justifies inequity and exploitation by accepting the existing order of things as given…This lack of critical judgment against society reflects an absence of what Cox (1974) refers to as “social ethics”; *it involves a subjective identification with an ideological viewpoint that admits no fundamentally alternative vision of society* (emphasis added, 1991, p. 135).

This last aspect of dysconsciousness is the primary obstacle for agents (individuals with privilege) to consider its implications.

### Grounding a social privilege process

How might one move from agent to ally in terms of social privilege? Although we sought to understand how an individual with a privileged social identity comes to be aware of it without assumptions about the course of their struggle, psychology sets a precedent for developmental identity processes that informed the authors’ training and practice leading up to the study. Identity development models related to the construct of social privilege have been published throughout the history of psychology. For example, in 1977, Ganter offered a three-stage model of White identity development. Later, in 2004, Spanierman and Heppner developed a theory of the psychosocial costs of racism to Whites. Perrin et al. proposed the transtheoretical model of behavior change for privilege awareness in 2013. With the lens of multiculturalism, Bennett created the developmental model of intercultural sensitivity in 1986. One of the most widely-cited models of privilege is a model of White racial identity development (WRID) of Helms (1984).

Nevertheless, there is a dearth of models within psychology especially that describe identity development for specific privileged identities outside of race or Whiteness. However, in 2012, Liu published the class and classism consciousness (SCCC) framework to capture the developmental process of individuals becoming aware of both their own and others’ socioeconomic status. The SCCC describes three levels of consciousness (no social class consciousness, social class self-consciousness, and social class consciousness) and 10 statuses (unawareness, status position saliency, questioning, exploration and justification, despair, the world is just, intellectualized anger and frustration, reinvestment, engagement, and equilibration). Liu’s model, adapted from its earlier iteration termed the Social Class Worldview Model–Revised (SCWM-R; Liu, 2001, 2002; Liu and Ali, 2008), is also noteworthy in that it also describes a spectrum of consciousness wherein individuals develop consciousness of class and classism as well as consciousness of their positionality as a “socially classed person” (Liu, 2011, p. 17).

Spanierman and Smith (2017) outline six steps that speak beyond White allyship and consider allyship in all social identity domains. One must:

1. demonstrate a nuanced understanding of institutional racism and White privilege;
2. enact a continual process of self-reflection about their own racism and positionality;
3. express a sense of responsibility and commitment to using their racial privilege in ways that promotes equity;
4. engage in actions to disrupt racism and the status quo on micro and macro levels;
5. participate in coalition building and work in solidarity with people of color; and
6. encounter resistance from other White individuals (Spanierman and Smith, 2017, p. 608–609).

While the final three steps are dedicated to engaging in specific behaviors, the first two foundational steps suggest allies should understand institutionalized privilege and oppression through continual self-reflection about their privileged social location. The authors thus argue that all allyship should begin with a fundamental development of social privilege awareness. They warn that without a commitment to this fundamental first step, even well-intentioned allies are susceptible to adopting “savior attitudes and behaviors.” In so doing the ally work can become shallow, fueling the privileged person’s desire to live in good conscience more than to enact and facilitate “deep structural change” (Spanierman and Smith, 2017, p. 610).

In pursuit of the APA guideline to recognize social privilege as a construct both broader than any single identity domain and applicable to practitioners and clients alike, we sought to understand the process of social privilege awareness within the individual. We were specifically curious how this awareness was born and progressed to the present moment. Our exploration was intentionally open-ended to allow for theoretical sensitivity (Glaser, 1978) and the active exploration of individuals from their perspectives of variously privileged social identities. We hope to fill this important gap with a theoretical understanding of how we integrate the thoughts and feelings of privilege awareness into our historic and contemporary identities.

## Materials and methods

### Research design overview

Because the process of initial awareness of social privilege and subsequent integration has not been distinctly defined, the authors used a qualitative approach with the intent for theory construction and further understanding (Stiles, 1993; Hill, 2012; Creswell, 2014; Richards, 2014). The concept of social privilege is by nature co-constructed between individuals within a context of overlapping social systems that establish and operationalize human hierarchy and caste. Thus, a social constructivist approach provided the lens to analyze the research question regarding social privilege integration (Charmaz, 2014). Considering the strong social justice implications of this project, a grounded theory approach encouraged the integration of researcher reflexivity, participant experience, and extant literature (Fine, 2013; Levitt, 2021).

Further, the authors believed the social constructivist and interpretive paradigm of grounded theory aligns with the most recent 2017 APA Multicultural Guidelines, which suggest “identity and self-definition are fluid and complex” (p. 4) and “developmental stages and life transitions intersect with the larger biosociocultural context…and these different socialization and maturation experiences influence worldview and identity” (p. 5). Specifically, classic Glaserian grounded theory provided a focus on theory construction and allowed for modification and change in the data analysis process (Glaser, 1978).

An individual semi-structured interview design allows for uniform questions regarding initial awareness as well as the unique developmental journey of each participant. Data analysis was conducted using a model of explicit power-analysis and power-sharing among the authors. The authors engaged different colleagues who were both familiar and unfamiliar with the aims of the project in an auditing process, providing for a diversity of inquiry and theory development.

#### Researcher description

As the qualitative researcher shapes the research results, it is generally important to practice transparency and clearly communicate pre-existing perspectives (Rennie, 1995; Morrow, 2007)—even more so in a project that explores social privilege and positionality (Harding, 1992; Hernandez et al., 2013). The authors engaged in this study as part of a larger research institute, established in 2017, focused on the exploration of social privilege. In preliminary literature reviews, the most interesting line of inquiry involved the initial awareness of social privilege into the individual’s identity and how it changed across time, setting, and relationship. Although each author is from a distinct social positionality, they share a common passion for understanding how social privilege operates. Prior understanding of the phenomenon included extensive exploration of the existing research as well as multiple presentations and classes on the general topic. Each researcher committed to practicing self-reflexivity in order to understand how social privilege has impacted identity formation, systems of attribution, and relationships.

The lead investigator is a middle-aged, middle-class, able-bodied, cisgender, heterosexual male of color that is a US citizen from a first-generation immigrant family. As a bi-racial individual growing up in the Midwest, questions regarding racial identity formation defined developmental tasks across his lifespan.

The second author is a white, middle-class, able-bodied, cisgender, heterosexual woman in her early 30s whose family has lived as US citizens for the last several generations. Narratives of the pain of privilege her mother experienced as a white woman growing up in the American South informed her identity development as she grew up in the relatively racially homogenous Pacific Northwest.

The third author is an upper middle-class, able-bodied, cisgender, straight woman of color that is a US citizen who was raised internationally. As a biracial and bicultural woman, she witnessed issues of power and privilege within her own family, tackling questions of belonging and colorism as she moved permanently to the United States. These informed her identity development and stages of privilege awareness.

Issues of power and privilege were explicitly discussed throughout the stages of this project. Following a feminist perspective, power was routinely examined and overtly analyzed with specific attention toward model construction. Both status and rank power were acknowledged, and modifications would occur *in vivo* (Nieto, 2010). Considering the project topic, the authors also examined their own positionality during the analysis process. Further, initial coding phases were conducted in pairs to aid with the exploration of how author positionality impacted the analysis.

#### Participant selection and recruitment process

Purposeful and snowball sampling were used in order to recruit a set of participants who identified as having agency (privilege) and who were willing to delve into a potentially difficult subject. Due to the nature of the topic, the team believed a general call for participants *via* listservs, flyers, etc., would result in a self-selection effect wherein volunteers would likely have already experienced initial awareness and some integration of social privilege into their personal identities. The authors, therefore, utilized their own social networks to recruit acquaintances who might have varying degrees of awareness and integration of social privilege. Of the 20 participants, 11 were clinical psychology students from a social-justice focused doctoral program in professional psychology and nine were acquaintances of the researchers and unaffiliated with the institution. Although doctoral students at the program were enrolled in a program with an explicit social justice mission, we did not assume this would be related to prior awareness of a privileged social identity. To the contrary, the authors’ experience suggested that participation in an organization (whether APA or the graduate program) that strives for social equity and justice does not necessitate understanding or awareness of the impact of any personal privileged social identity. Additionally, we assumed all participants, regardless of student status, would have some experience of becoming aware (or not) of a privileged social identity, which we aimed to understand. Given the personal, professional, and collegial relationships with participants, the team made efforts to reduce and minimize undue harm and influence. Participation in the study was strictly voluntary and there were no negative consequences for non-participation. Given that the first author is a professor, interviews with student participants were conducted only by pre-doctoral students while participant names and identifying information were removed from data collection and transcription of interviews.

Given that the research was focused on participants’ experiences of social privilege, in lieu of a questionnaire that collected specific demographics of each participant, a questionnaire based off of the ADDRESSING model of Hays (2016) was used and participants were asked if they had privilege in each ADDRESSING domain (age, developmental/acquired disability, religion, ethnicity/race, socioeconomic status, sexual orientation, indigenous status, nationality/citizenship, and gender identity/sex assigned at birth). According to the model, social privilege applies to people who are age 18–65, identify as not having a developmental/acquired disability, identify as a Christian/cultural Christian, are ethnically and racially White, have a middle/upper socioeconomic rank, identify as having a straight/heterosexual sexual orientation, do not have indigenous status, have American nationality/citizenship, and/or identify as cisgender or have a male gender identity. The limited specificity and detail of this questionnaire protected the privacy of participants who were acquaintances of the researchers. All participants had at least one privileged social identity domain, thus no participants were excluded from participation based on their responses to this questionnaire.

Once potential participants were identified, they received an email detailing the study along with an IRB-approved consent. No incentive or compensation was offered. Participants were chosen in order to obtain as much diversity of privilege in identity domains as possible. In order to increase the comfort of participants and promote an open dialogue, researchers conducted interviews with participants they were acquainted with. Since there were three authors, interview coding was conducted by someone who did not conduct the interview and did not know the interviewee; this also helped to reduce bias and increase objectivity in analysis.

Inclusion criteria for the study permitted only individuals who identified as having privilege in at least one social identity domain as assessed by the ADDRESSING questionnaire, were 18 years or older, were willing to talk about their experiences of social privilege, and read and signed the informed consent. Two of the 20 interviews were excluded from the data set due to various reasons; one potential participant was under age 18 years and one interview was inaudible. Interviews stopped at 18 participants due to theory development and saturation. Glaser (1978) describes saturation as the point at which a developing theory’s explanatory power reaches a critical point. Comparison of the theory at that point to further data meets the criteria of work, fit, and relevance while the new data did not substantially modify the developed theory.

### Data collection and grounded theory analysis

Two forms of data were used. Analysis focused first on semi-structured, open-ended interviews collected in alignment with the grounded theory methodology. Interviews started with a “grand tour” question inviting the participant to generally dictate the content, direction, and process of the interaction. A standard definition of social privilege from three sources (Google Dictionary, a local group of scholars, and McIntosh, 1989) were provided upon participant request or when the interview determined it was useful.

As the first round of interviews was completed, parallel data analysis pointed to general questions regarding initial awareness and sequential progression. The interviewer could follow their curiosity and adjust the inquiry as the interview developed. Interviews were conducted in comfortable, private locations, mostly in person (16) and some over the phone (2). The average interview took about an hour. Interviews were audio-recorded, transcribed, and anonymized manually by the interviewers. Transcripts were then transferred to the data-analysis software Dedoose, and then the recordings were deleted.

In addition to the interviews, eight students also consented to having their written response to a course reading (DiAngelo’s, 2016 *White fragility: Why it is so hard for White people to talk about racism* and *A people’s history of the United States* of Zinn, 2003) coded for additional data in order to check the saturation of the theory following the analysis of the interview data. Comparison of these responses to the theory that had emerged from the 18 interviews yielded no new themes. The fit of the written reflections with the theory at that point and the work of the theory to explain the process captured in these reflections indicated saturation of the theory.

The team used grounded theory analysis (Glaser, 1978) focused on the participants’ struggle of coming to terms with social privilege, how they conceptualize the struggle, and their attempts to resolve said struggle. In grounded theory, analysis usually occurs directly following the first interview and proceeds in parallel with data collection. Memoing incorporates researcher reflexivity and further informs the data collection and analysis process. Analysis begins with open coding, in which the researcher looks for indications of the primary struggle. Open codes are then condensed, based on similarity and common themes, to categories of conceptual codes (axial coding). In exploring the relationships between these concepts, and paying attention to how the participants attempt to resolve the struggle, core variables emerge (selective coding). Throughout the process, the researcher uses theoretical sampling to identify theoretical constructs that have emerged in the data, and then attempts to apply the concept to all the data. This leads to an iterative process of constant comparison in which established concepts are interrogated using new data in order to test veracity. The grounded theory is constructed from these core variables and attempts to explain how they fit together.

Grounded theory uses four criteria to determine its analysis: fit, work, relevance, and modifiability. Data are not forced into a pre-existing framework, but instead fit together to describe the experience or process of the participants through overarching categories. Data that remain are used to modify existing categories at later stages in the analysis using constant comparison (Glaser, 1978). The resultant theory needs to follow the principle of work by explaining the struggle, the attempts toward solutions, and the overall process of the phenomena in question. A grounded theory has relevance not only for the participants of the study, but also others who are involved in a similar social process. Thus, the theory is not a representation of the specific data set, but rather a conceptual framework with explanatory value for anyone involved in a similar struggle. A grounded theory is never finished, as it must be modifiable in the face of new data from any source. This is distinct from the “prove or disprove” interrogation of the hypothesis in positivistic quantitative approaches. Although our theory reached a point of saturation in the last few interviews, in light of modifiability, it is constantly subject to change.

The researchers employed the principle of fit through line-by-line analysis of each interview and incorporation of all codes into categories that would later make up the themes of the growing theory. We applied the principle of work by checking that the growing theory worked to explain the struggle of each participant. In service of these two principles, the researchers read and re-read each interview several times. The developing theory grew around the experience of a privileged social identity, rather than any status as a student, practitioner, or other professional role, which varied across the sample. We engaged the emerging theory in the principle of relevance through self-application and discussion with colleagues who concurred with the theoretical outline we could sketch at that point. These discussions also honed our understanding of the struggle of the participants. To check for the principle of modifiability, we both held the theory up to the written reflections of eight students in the graduate program and, conversely, those reflections up to the theory, which yielded no new categories. Although we took this as a sign of saturation for the current study, we hope future studies will modify the theory regarding the relevance of Model of Integrating Awareness of a Privileged Social Identity (MIAPSI) to more specific groups, either within or beyond the field of psychology. These studies might take a more pointed approach to understanding MIAPSI longitudinally and cross-sectionally.

Another guiding characteristic of grounded theory methodology is the parallel deductive and inductive processes of analysis. The research team used simultaneous interviewing and analysis in an iterative process to begin developing core categories and concepts—an inductive process—that could be further investigated during subsequent interviews—a deductive process. The team conducted one interview each, transcribed them, and completed an open coding analysis. Then the team shared their experience of the interviews, their impressions, and the open codes. Fellow team members would review the transcription and open codes, adding their own codes and discussing the similarities and differences. Once the interviews and open coding team sessions were complete, the team progressed to axial coding and defined core categories, shifting from individual to group analysis. Then, the team began conceptual analysis, exploring how these categories fit together in terms of sequence, pattern, and quality.

#### Methodological integrity

Analysis and open coding of the interviews occurred in pairs (the authors and other lab members) to establish consensus process. From this initial stage, members began to hone their interview skills through debriefing; they also developed a further understanding of the coding process that assisted in identifying further lines of inquiry for future interviews. The next stage was a simultaneous process of interviewing, transcribing, and open coding using paired coders. During this process, analysis work occurred both during and outside of meeting times. Memoing was consistently being completed, shared, and integrated to address researcher reflexivity (Levitt, 2021). The three authors established a general consensus process in which any part of the findings was agreed upon. Findings were grounded in the evidence as the open codes, and conceptual categories were derived from the nexus between the interview data and the authors’ interpretation of the data informed by their social positionality and ongoing conceptual inquiry. This took the form of ongoing author memoing, analysis meetings, and external auditing with other lab members. A stage-wise developmental framework emerged from this process and new interviews began to generally fit within this framework with little modification, signifying saturation. Fidelity to this grounded analysis process was maintained throughout, with no alterations in the analysis process for methodological or ethical reasons.

Researcher perspectives were considered a viable part of the dataset and were discussed and documented in various meetings. Lab meetings consisted of data analysis in which the authors processed their thoughts and reactions, and then integrated them into the analysis process. This helped to develop additional lines of inquiry and focal points for upcoming interviews. Further, the meetings would often include outside lab members to provide a fresh perspective and reactions that assisted in further inquiry identification and theory development, constituting a type of auditing process (Levitt, 2021).

## Findings

**Figure 1:**
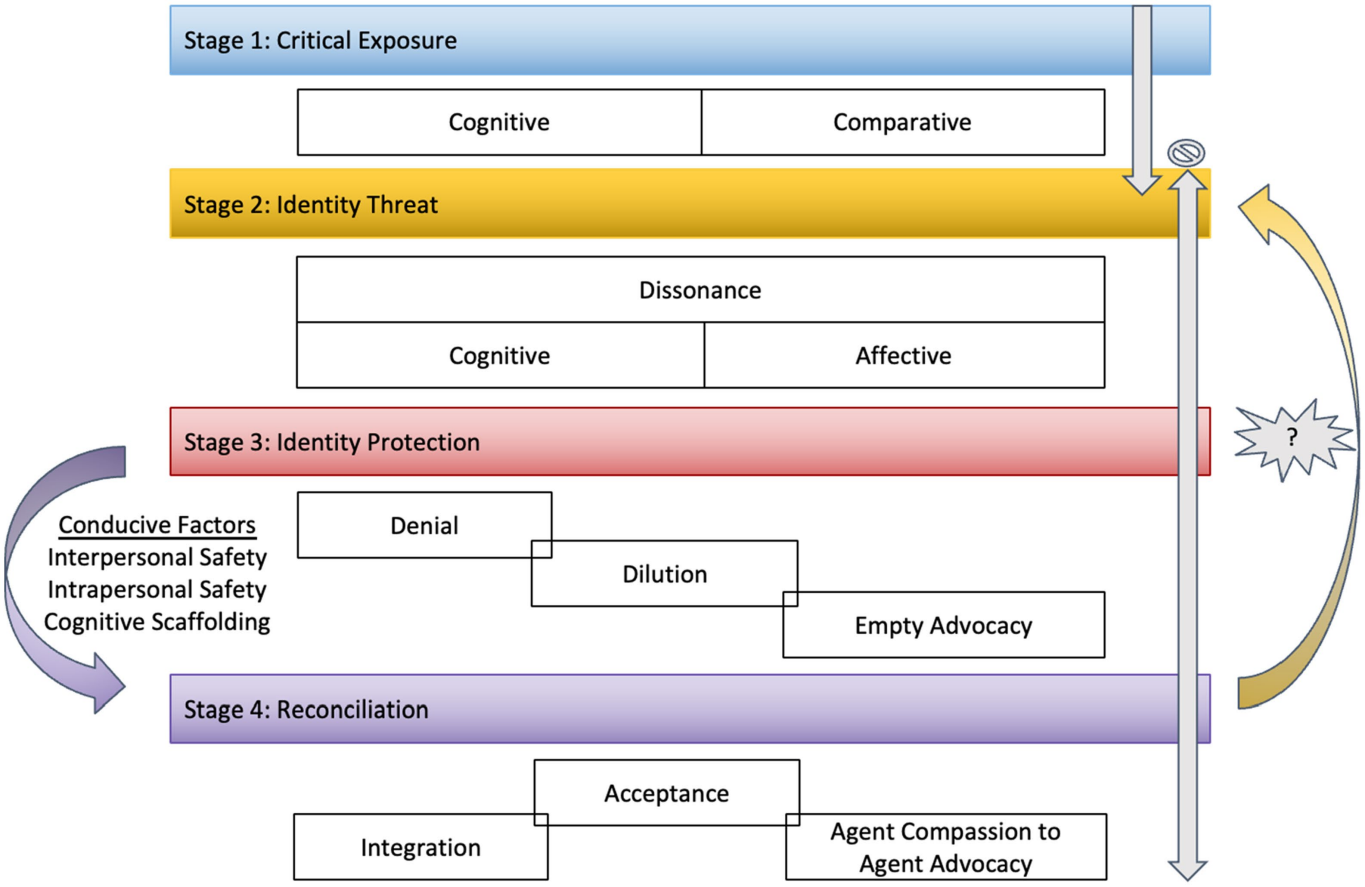
Model of Integrating Awareness of a Privileged Social Identity (MIAPSI).

Upon the distillation of codes and categories, a developmental process of social privilege awareness emerged with four developmental stages: (1) Critical Exposure, (2) Identity Threat, (3) Identity Protection, and (4) Reconciliation (Figure 1). Each stage is characterized by distinct experiences. The first stage, Critical Exposure, is characterized by gaining a new or renewed awareness of social privilege through both Cognitive and Comparative Exposures. The second stage, Identity Threat, is distinguished by Cognitive and Affective Dissonance, while the third stage, Identity Protection, involves strategies of Denial, Dilution, and Empty Advocacy, which aim to eliminate or minimize the dissonance. Finally, the fourth stage, Reconciliation, is characterized by experiences of Acceptance, Integration, and Agent Compassion to Agent Advocacy in which the person seeks to resolve the dissonance of social privilege. The researchers also defined conducive factors (including Interpersonal Safety, Intrapersonal Safety, and Cognitive Scaffolding), which help to create a fertile space for social privilege awareness development.

### When did social privilege awareness start? Stage 1: Critical exposure

Stage 1, Critical Exposure, is the beginning of an individual’s journey in social privilege awareness. The stage is characterized by an impactful moment in which awareness of privilege, in any social identity domain, burns through dysconscious barriers and into clear and undeniable focus. Participants commonly recalled being exposed to privilege differences multiple times throughout their childhood, adolescence, and adulthood. Yet, most of these exposures did not result in a critical exposure. Homogeneity of values, customs, and relationships fueled the internalization of privileged hegemony (White, male, able-bodied, cis-gender, etc.) which in turn rigidly maintained dysconsciousness. The repeated exposures participants described demonstrated the power of dysconsciousness to withstand evidence of inequity and to preserve a state of privilege unawareness throughout one’s life.

Nevertheless, Critical Exposure can be viewed as an accumulation of exposures that builds fuel for the eventual ignition of privilege consciousness, eventually burning away the veil of dysconsciousness. Sources of exposure include the media (news, TV and movies, books, and social media), education or cognitive exposure (classes, lectures, professors, and peer discussions), and interpersonal relationships (classmates, teammates, friends, and family), as well as temporary relationships formed through travel or volunteering. This critical consciousness commonly occurred in early adulthood, when the participant was already exploring their identity, seeking individuation from their social system of origin, and was less likely to have a family of their own. This independence helped individuals endure the social collateral damage that inevitably followed critical consciousness. The scorching realization of racist or sexist grandparents, parents’ classist assumptions, or friends’ xenophobic ideologies created wounds of critical exposure.

Critical Exposure cannot be reversed or undone. It is the cognitive leap from various and obscure encounters with another person’s experience of oppression to the individual’s recognition of systemic oppression. The participant’s application of this insight to their own life and experience required additional cognitive sophistication and intrapersonal stability, and seemed to initiate effective reflection on anti-oppressive action. The data indicated that participants needed both cognitive and interpersonal (comparative) exposure to make these leaps; the resultant understanding of social privilege varied in sophistication, depth, and complexity.

Another important aspect of Critical Exposure was the individual’s perceived sense of volition in the experience. Some participants decided to read a book, watch a movie, attend a lecture, or ask questions of diverse friends to satisfy a cultural curiosity. Other participants discussed taking a required multicultural class in school or a diversity training at work in which they were exposed to privilege differences. When individuals appeared to have a choice in exposing themselves to experiences of critical consciousness and act from their own motivation, a critical exposure was more likely to occur with ease. However, when individuals felt forced into these experiences, the revelation of critical consciousness was not always welcomed. Thus, volitionality was found to be a variable factor that could predict the development of or resistance to social privilege awareness.

#### Cognitive exposure

Cognitive Exposure is the intellectual introduction to the concept of social privilege. Usually originating from education or media, it is more passive than comparative exposure. This modality appeared to engage individuals in a primarily cerebral and private space, shielded from the public display of uncomfortable emotions or the judgment of others, thus softening the exposure and decreasing the likelihood and the sting of overt privilege awareness. For example, Participant 9 recalled their Cognitive Exposure:

My experience I guess was historically ignorant? I was like, “what privilege?” You know, a lot of the stereotypical or cliche lines I guess that you hear out there… but, I think over the course of the last 10 years, I think I can trace it back to my undergraduate critical thinking class. And it was like starting to challenge some of my ideas, and I think it helped.

In some cases, repeated cognitive exposures could be interpreted as slowly aggregating into critical awareness. Or, Cognitive Exposure at a particularly incendiary moment synthesized all prior exposures that did not quite catch fire.

#### Comparative exposure

Comparative Exposure moves beyond the intellectual concept of social privilege to the interpersonal. It involves being exposed to other people who had differing domains of social privilege, thus eliciting a self-consciousness of the participant’s own social location. Usually, participants recalled witnessing or hearing stories of friends and family being discriminated against, which elicited thoughts relating to systemic oppression. Another participant, who identified as an athlete, discussed realizing their able-bodied privilege through traveling and interacting with athletes who have disabilities. Two participants realized their White privilege through their friendships with Black peers. Another referenced stories they heard from their mother who grew up in a developing nation and low-SES family. From these striking moments of interpersonal comparison within their own current and past life experiences with others, participants acknowledged power differences and attributed them to societally defined identity ranks. For example, one participant described the context in which they experienced comparative exposure:

I lived abroad and a lot of those places where I’ve lived are third world countries. And so, just living in those places and knowing that you drive in a car while there’s kids outside asking for money, it’s hard to deny that you’re on an even playing field.

Another participant on the other hand, described their own process of Comparative Exposure:

Because as people we constantly compare ourselves to others, right? And when you’re constantly surrounded by people who are maybe like the same socioeconomic level as you and live in a suburban neighborhood, you know? You’re kind of similar. So, how are you going to say that someone is more privileged than you until you’ve actually seen others that don’t have the same quality of life? Then… you can see that the group and level you belong to is also privileged. So, I think it starts at the personal and experiential level.

In these cases, a compassionate and empathetic human connection prevented a negative attribution to the individual, and social identity emerged as the only other possible explanation for the disparity of experience. This comparative process broke through stereotypical ideas of meritocracy and value judgments, and demanded that the participant question, for example, “why do bad things happen to my friend who’s a person of color and not me? How did I not experience the challenges that I see my female partner experience every day?” This active comparison created space for awareness of social privilege that had not previously been considered. Further, due to the emotional salience of interpersonal relationships, comparative exposure was more powerful than cognitive exposure; it seemed to result in greater commitment to reconcile social privilege.

It is important to note that insight into social privilege was secondary to the focus on oppression in both types of exposure. Participants normally started with a story about how others are discriminated against, and then they associated insights regarding how this discrimination was supported by societal mechanisms. Moralistic judgment characterized these realizations of the reality of oppression, which in turn appeared to elicit feelings of sympathy and outrage, as well as the assumption that the participant was an observer in the oppressive process. Yet, somehow, the realization of oppression drew the participants to reflect on their own resources, hardships, and accomplishments. Through exposure they came to understand that they have been saved from certain challenges simply due to the circumstances of their birth.

### What is the impact of social privilege awareness? Stage 2: Identity threat

The critical awareness of social privilege as a direct corollary to oppression can be highly disruptive to an individual’s identity. Social privilege calls into question almost every domain of personhood, from relationships and accomplishments to morals and values. After participants recalled their Critical Exposure to the concept of social privilege, almost all reported a sense of deep incongruence within their self-understanding. Identity Threat occurs because the concept of social privilege is experienced as inherently dangerous; it therefore threatens the autobiographical stories and memories a person has collected to create the identity narrative that provides cohesion and continuity of self.

The prominent risk of social privilege awareness is the loss of our internalized image of ourselves. The gradual attributions regarding our accomplishments and relationships are ingrained from a hegemonic social system starting at birth. A social system is hegemonic when the ideologies that naturalize it ensure subordination to and acceptance of the stratified social order (Lewis, 2004). The concept of privilege reveals alternative ideologies and explanations for stratification and calls into question the etiology of our life trajectory, from the circumstances of our birth family, our influences throughout childhood, our intimate relationships, and our professional pursuits. Social privilege directly threatens the assumption that our circumstances are due to our volitional actions and connections. Instead, it points to the possibility that we are partly who and where we are due to the societal conferral of privilege through an oppressive, colonial system of distribution based on our externally-perceived identity domains. The data indicated that the most common source of dissonance was around meritocracy, causing the individual to question whether they were directly responsible for their accomplishments or if social privilege was involved. In response to this threat, participants asserted that they too suffered from hardships and setbacks as a way to minimize the inherent privilege in their social domains.

Results suggested social privilege awareness interrogated fundamental American ideologies, such as meritocracy, freedom, rugged individualism, equality, and the belief that privileged people are unquestionably virtuous. Accepting the concept that social privilege exists requires the individual to modify their identity narrative, to allow the concept to literally edit the story of their past, present, and future. The resounding quality of resultant Identity Threat triggered by critical exposure was a sense of incongruence between previously held beliefs, ideas, and values and the impending modification that would occur if awareness was fully conscious and integrated into intra and interpersonal life.

Another important aspect of this stage is the contradiction between an individual’s internal versus external identity. In most cases, participants did not like to be associated with privilege, as it was felt to minimize their individuality. For example, most White participants did not like being labeled as White, as it was stereotypical and did not acknowledge all the other aspects of their personhood. This internal/external contradiction could also be perceived as a type of “reverse-racism” or other—ism, as it placed the individual in a clumsy, socially-constructed box. Yet, not having this contradictory feeling in everyday life could be a strong indicator of social privilege possession itself. Participant 15 alluded to this in a written reflection:

In DiAngelo’s construct of… “new” racism… I’m supposed to identify my Whiteness with a feeling that I’m “smart, deserving, and capable” and that I can effortlessly take over in social settings with people of different races. In actuality, I’m a woman, I’ve been socialized not to be so overbearing and assume my point of view is the one that always needs to be heard, so I reject that premise that reduces me to a racial stereotype.

Participant 8 also reflected on their internal versus external identity as they stated:

The role that perceived identity has, I think we all like to think… you’ve had these experiences, and this is what you call yourself and who you are, but the perceived identity, obviously… can be the dominant identity that is influencing how people treat you.

A final considerable risk to allowing the concept of social privilege into our conscious awareness is the palpable danger to our past, current, and future relationships. Considering the deep permeation of the concept into the structures of the self, the consequence of awareness would also create conflict and rupture in relationships with others. If we acknowledge our privilege, it could cultivate the question as to why our parents and family did not discuss this possibility in the past. This basic questioning could be perceived as disrespectful and threatening to the family and wider social network.

#### Cognitive dissonance

Cognitive dissonance, as proposed by Festinger in 1957 (Harmon-Jones and Mills, 2019), motivates an individual to avoid information that increases the incompatibility (dissonance) between two thoughts or ideas or otherwise reduce the discomfort of that dissonance. The parallel realization of the oppression of others and the unearned possession of social privilege causes confusion and Cognitive Dissonance. An individual’s values and beliefs underlying previous dysconsciousness suddenly conflict with the emerging awareness of privilege. Participants described a *paradox of privilege*, in which participants wanted to rid themselves of social privilege because they had begun to comprehend their ancestral and historical bond to systemic oppression; a realization which inevitably threatened a person’s identity narrative and caused them to feel as though they are a “bad” person, or even personally responsible for systemic oppression. This realization also put their familial relationships in jeopardy. Paradoxically, however, while they experienced danger in their social privilege, individuals also wanted to hold onto their social privilege because they recognized it provided systemic advantages and benefits. This paradox of privilege was further complicated by the fact that individuals cannot be absolved or liberated from social privilege. Participant 1 described cognitive dissonance as she wrestled with the paradox of privilege:

So… you want candid? We’re comfortable and I wouldn’t change that and I mean, you want to talk about talking out of two sides of your face? So… I want a just world, I want that. I wouldn’t want to sacrifice, you know, my children’s comfort for the greater good, I guess? Is that what I’m saying? I don’t know!

#### Affective dissonance

The prospect of resolving dissonance between the implications of a privileged social identity and an identity narrative as a just and moral person after Critical Exposure leads also to emotional discomfort, or Affective Dissonance among seemingly incompatible feelings. Affective Dissonance was characterized by the uncomfortable and internalized feelings of guilt or shame upon awareness of the benefits and advantages of social privilege. Participants described it as “that icky feeling of not recognizing you are benefiting from something” and feeling “gross and dirty” after awareness.

### What is the knee-jerk reaction? Stage 3: Identity protection

The combination of cognitive and affective dissonance drove participants to rely on dominant hegemonic concepts and beliefs to self-soothe and hedge the perceived risks of privilege awareness. Each participant’s attempt at protection was idiosyncratic and influenced by their personality, identity narrative, and inherited worldview. Yet they all had the same end goal: to nullify the threat of awareness and critical consciousness. Some did this by lashing out at oppressed groups and claiming their own personal struggles and hardships were comparative, or by intellectually critiquing the very existence of privilege and its corollary, systemic oppression. Alternatively, and even more confusing, some participants attempted to bypass their own privilege awareness by aligning with oppressed groups and helping those in need. These strategies are often automatic and unconscious responses to Identity Threat and include a unique combination of affective, cognitive, and behavioral correlates. Three distinct strategies were identified and labeled Defense, Dilution, and Empty Advocacy.

#### Defense

Defense is a protective strategy that is characterized by high emotionality while cognitively relying on the hegemonic views that scaffolded previous dysconsciousness. This strategy seemed to immediately occur in direct reaction to Identity Threat, in which the perception of threat was at its highest. It appeared that soon after an individual had inkling as to the implications of privilege awareness (revision of their self-concepts and risks to relationships), a flood of aversive fear, shame, and anger pushed them away from the sources of exposure.

An impulsive stream of thoughts often accompanies the emotionality of Defense, which aims to deny the existence of social privilege, the superiority of agent ranks, and the inferiority of target ranks. These thoughts usually highlight meritocracy, superior work ethic, cultural heritage, and moral standards for agent ranks and the lack thereof for target ranks. The source of these thoughts appeared to be socialized beliefs that maintain the existing power structure with the aim to comfort individuals with prominent agent ranks. Participants reported that these thoughts were associated with strong feelings of fear, shame, and anger, but rarely admitted that these were overtly expressed to individuals with clear target ranks. More commonly, agents appeared to share defensive thoughts with other agents who had also experienced a Critical Exposure without a prior understanding of the impending threat.

Compared to other Identity Protection strategies, Defense appeared more reactionary and confusing versus fully intentional and volitional. Also, Defense appeared to be the more difficult strategy for participants to articulate, possibly due to the concomitant guilt. While this does not nullify personal responsibility of agent behavior, it was important to consider that part of the etiology of this behavior was in reaction to Identity Threat.

When experiencing Defense, an individual demonstrates feelings of fear, guilt, and anger which can often manifest as overt or covert expressions of racism, sexism, classism, homophobia, or other -isms. An overt expression would be clear interpersonal violence or discrimination; a covert expression would be a microaggression of which the aggressor is “unaware” (See Sue et al., 2007 and Neville et al., 2013 for implications of microaggressions in clinical practice). Defense might also involve victim-blaming: in an effort to divert responsibility, an individual uses oppressed persons or groups as a scapegoat for their own reactive emotionality.

#### Dilution

Dilution involves cognitive strategies which question the veracity of the social privilege construct, mainly by utilizing personal experience as the referral point to judge new information. Dilution is different from denial in that the individual might pay some heed to target rank experiences, and maybe even recognize some systemic and social discrepancies, but ultimately will critique the existence of their own privilege conferral as the byproduct of oppression by others instead of direct social benefits they have received. Instead, participants will claim they have not experienced clear advantage at the systemic cost to others. They will pose questions with the intent to invalidate target experiences of others, the reality of systemic oppression, and their own benefit from the system.

The data indicated participants used multiple tactics to dilute social privilege, including demanding proof, establishing subjectivity, and confusing status and rank. Participant 7 shared their experience of Dilution as they explained:

It’s not like I’m a privileged person or I am not a privileged person. It’s that there are all sorts of different characteristics or dimensions that you can have more or less privilege on. So like, I’m White, so that would be a privileged position. I would also say that at least in a theoretical sense, it depends on the context. And, in our world, being White is pretty much always going to be the privileged position but there are contexts in which things might be, like, more or less.

Dilution begins with demanding undeniable proof of oppression and privilege, taking advantage of the dysconscious and elusive nature of privileged socialization. A person using Dilution positions their own life experience as the criteria for validating oppression’s existence. Dilution seeks an external source to prove privilege in the light of agent experience, which is practically impossible. This also puts the burden of proof on the target experience and other advocates instead of on the agent.

Next, Dilution attempts to establish the subjectivity of target experiences, oppression, and privilege. Upon encountering alternative perspectives, agents can claim that multiple other factors are involved, resting on the idea that we are all unique and different. Further, any attempt to group individuals in order to exemplify social discrepancies is met with accusations of inaccurate stereotyping and possible “reverse racism” (and other -isms). In addition, in contrast to the high emotionality of Defense, results suggested people protecting themselves through Dilution stay in the cognitive realm: they intellectualize social privilege to distance themselves from the impact of fear, guilt, shame, and anger.

#### Empty advocacy

Empty advocacy involves retreating into efforts that appear to help those less fortunate, but is devoid of privilege awareness that is integrated into the individual’s identity narrative. It usually takes the form of volunteer work (food banks, women’s shelters, construction projects, etc.) that are socially assumed to directly assist target groups; it can also take the form of helping friends and family. Empty Advocacy efforts need to be directed toward less-fortunate individuals and, fundamental to the strategy, witnessed by others in order to make the agent appear as though they are further along in their privilege awareness development. The efforts usually occur in a manner that does not draw overt attention to the individual’s privilege, and maintains the congruence of their identity narrative. Although the behavior may appear to be the same as Agent Advocacy, it lacks a clear understanding of power dynamics or overt intentionality; instead, it tends to be directed toward assisting target populations without an effort to change agent perspectives and systemic oppression. Participant 8 described a moment of clarity when they realized their past engagement in Empty Advocacy:

Fast forwarding to when I have a recognition of "hey, maybe I have some privilege too," was in college, getting involved with a student organization that does community service events…. I participated in a training where we brought in like a professional consultant, facilitator, around multicultural issues, and she asked some really great questions, put us through sort of a discussion process, where she had us describe why we do community service. And then really the pivotal question that sort of, for me, was very influential in how I thought about myself and what I do for community service was the question of "when you do community service, who benefits the most?" and the recognition that like "wow," to date, a lot of things that I’ve been doing, the person who benefitted the most was probably me, not the person that I was serving.

The action involved in Empty Advocacy is thus a strategy for agents to distance themselves from the threat and dissonance of social privilege, as well as bypass the difficult work of integrating social privilege into their identity. Acting thus can serve to convince both the self and others that the problem of innate privilege has been solved.

Empty Advocacy can assuage the fear, guilt, shame, and anger that result from privilege awareness. It can suppress the intellectual struggle of questioning the dominant hegemonic values that previously shaped individuals’ identity, and it can be used to deflect perceived accusations of bias by others. When issues of oppression and privilege are conflated with individual acts of discrimination, Empty Advocacy is effective in shifting the accusations: someone could not be, for example, racist if their volunteering benefits BIPOC individuals. Empty Advocacy can also support someone’s virtuous image of themselves, allowing them to remain blameless in unintended harm and microaggressions. Motivation to protect oneself from the threat and dissonance of privilege awareness is foundational to empty advocacy.

### What does it take to progress? Pre-stage 4: Conducive factors of reconciliation

Identity Protection, in its three forms, was a common sticking-place where participants would cycle between emotional and cognitive avoidance and misinformed helping behaviors. Participants seemed to describe or depict this cycle in the present moment, meaning that most were currently embodying Stage 3. The next inquiry focused on the participants that seemed to have moved beyond Stage 3, and more specifically, how this shift occurred. From this, four conducive factors appeared to foster a move toward identity narrative revision in light of social privilege, or Reconciliation. The data and emergent theory captured four conducive factors that facilitated participants’ movement to Reconciliation: (a) Interpersonal Safety; (b) Intrapersonal Safety; and (c) Cognitive Scaffolding.

#### Interpersonal safety

Psychology posits that we are social beings and that we know and define ourselves in relationship with others (Fiske, 2004). Thus, our social relationships define us and can either keep us stuck in a current state or help us grow and change. An important conducive factor that appeared to foster the integration of social privilege awareness and identity narrative revision was Interpersonal Safety—an experience of emotional and relational safety within supportive relationships. Safe relationships, which include space for honest dialogue with low risk of retaliation or loss, help provide fertile ground for social privilege awareness. Without Interpersonal Safety, the cost of identity change and explicit ownership of social privilege can be too high.

Interpersonal Safety can derive from relationships with individuals holding both agent and target ranks, with each providing distinctive support. Relationships with agent allies—others that share participants’ agent ranks—appeared to be the most conducive promotion of identity narrative revision. Agent allies are persons who have developed social privilege awareness and can guide other agents through the developmental stages of awareness. They can provide mentorship or valuable insight into their own developmental process, ensuring that Stage 2, Identity Threat, does not have to be a permanent experience and that Stage 4, Reconciliation, can be healing. The agent ally provides comfort, knowledge, and security while modeling a process that is inherently confusing and uncomfortable. For example, a man who is coming to terms with sexism and his sexist privilege can benefit most from relationships with other men who also are engaged in the revision of their own identity narrative. White individuals stuck in Stage 3, Identity Protection, can similarly be supported by White friends who have moved beyond protection and can serve as examples or even mentors. Participants who reported these kinds of relationships were most likely to move from Identity Protection to Reconciliation.

Target relationships can also offer avenues toward Reconciliation. The proximity and valence of a target relationship mediates safety within the relationship. This can have an additive effect for an individual with social privilege in a caring relationship with a target person: the agent can become more aware of the ways in which the target is treated differently or unfairly, thus assisting in the agent’s self-reflection and development of social privilege awareness. Target relationships often provide the motivation to increase social privilege awareness *via* the agent’s desire to not inadvertently cause additional suffering for close targets.

Social privilege is conferred from systemic sources of hegemonic values, beliefs, and customs that are collectively established and manifested in our daily lives. This power of pervasiveness can be too strong for an individual in isolation to challenge and redefine. Utilizing social interaction and support to dismantle supremacist hegemony, the same mechanism that established social privilege in the first place, is the most effective way to support personal change.

#### Intrapersonal safety

A sense of stability within the core identity supports an agent’s development of social privilege awareness. Given that social privilege threatens agents’ identity narratives and belief systems, agents without a flexible core identity, resilient in the face of dissonance, can become stuck in Stage 3. Some individuals take the “off ramp” of denial for a quick exit from dissonance, returning to the relative comfort of various myths that increase congruence of their identity narrative with the dominant social order. A flexible identity narrative can withstand the integration of social privilege and an inevitable transformation. Intrapersonal Safety also helps agents tolerate the many tensions such as the paradox of privilege and the drive for continued anti-oppressive action for change, despite uncertainty that change will occur.

Intrapersonal Safety additionally cultivates opportunities for experiential empathy. Experiential empathy occurs when a person with intersecting privileged and oppressed social identities draws from and compares personal experiences. As a hypothetical example, a man who identifies as Asian-American can reflect on instances in which he experienced institutionalized oppression through racism. He can reflect on his shame, sadness, fear, and anger in these transgressions and empathize for his oppressed identity while also reflecting on his privileged identity as a man. This juxtaposition can encourage him to realize he does not want to oppress others as he has been oppressed. Experiential empathy requires Intrapersonal Safety because there is dissonance and discomfort in recognizing one is both part of an oppressed group and part of a privileged group, creating a “loyalty pull” between the identities.

#### Cognitive scaffolding

Cognitive Scaffolding helps an individual to (1) translate discomfort into awareness of structural oppression and privilege and their material outcomes; (2) normalize their discomfort; and (3) strategize for positive change. Vygotsky (1978) originally described cognitive scaffolding in relation to the zone of proximal development in which a person learns a new skill with support from someone with more experience and knowledge. Within a Cognitive Scaffold, individuals with social privilege can organize and make sense of new ideas and anchor their emotional reactions to social privilege awareness. Cognitive Scaffolding can, therefore, be particularly helpful and effective for agents who have a salient Affective Dissonance response to social privilege awareness. Examples include definitions, facts, well-researched and trusted theories, developmental identity models, personal experiences, and an accurate and nuanced history of systemic power, privilege, and oppression.

### Stage 4: Reconciliation

Reconciliation entails an individual’s effortful integration of their new awareness of social privilege with their existing identity narrative. The individual thus emerges from this stage with a newly modified identity narrative and sense of the world that integrates their understanding of social privilege. The individual also emerges with a more developed and sophisticated comprehension of social privilege and a more nuanced approach to advocacy.

Our emergent theory indicates there are four core experiences involved in an agent’s reconciling the complexities and conflicts of social privilege and the integration of an understanding of social privilege into their identity narrative. These Reconciliation experiences include: (a) Acceptance; (b) Integration; and (c) Agent Compassion to Agent Advocacy.

#### Acceptance

Reconciliation entails acknowledging and accepting social privilege awareness, a state which intrinsically involves vulnerability and unease, and which may include cycling through Stages 2 or 3 in the course of acceptance. Acceptance involves acknowledging that privilege cannot be resolved or purged, but instead needs consistent tending and acknowledgment. In addition to feelings of shame or guilt, there is an accompanying confidence that acceptance of social privilege can coexist with a compassion for self and others. A person in Stage 4 might recognize their social privilege does not define them or make them an inherently “bad” person. They may recognize that although they are a part of systemic oppression, it is not their fault as an individual. Furthermore, although the person recognizes the structural and material outcomes of social privilege are abhorrent, they find comfort in knowing that they are on the path to change. For example, participant 6 shared:

I’m starting to move out of guilt. It’s been a lot of guilt. And frustration, but I’m starting to recognize that it isn’t through my personal actions that I was put in this socioeconomic status… but I can choose what I do with my position. And so, I’m kind of coming to terms with that.

This recognition that a privileged social identity is not chosen and provides a platform for justice echoes a description of White allyship from Williams et al. (2022). Participant 18 also expressed Acceptance as they stated:

For me… it just kind of felt… we talked about a bunch of other privileges that… it made me feel better. Like I still feel bad but it made me feel, like, it’s okay. Like it’s not my fault.

#### Integration

With new awareness, individuals can integrate the paradigm of social privilege into their existing identity narrative as part of Reconciliation. A person allows the identity narrative to change and seeks new opportunities to further deepen their awareness. For example, a participant shared their Integration of the fact of social privilege into their way of being as they described their next steps:

I think I’m just starting to understand what we do, and I think the first thing is admitting there’s a problem. That’s step one, is acknowledging these systems exist and they are hurting people directly, and their pain is real. Their experiences are real and that each of us, maybe in small ways, maybe in big ways, maybe actively, maybe passively…but we’re all contributing to it in some way or another. People of privilege are contributing to oppression in one way or another.

This participant demonstrates they have integrated the concept of social privilege awareness into their identity narrative by describing and acknowledging their part in systemic oppression.

#### Agent compassion to agent advocacy

Reconciliation additionally involves an ability to have compassion for other persons’ social privilege awareness process. Through this compassion, an individual demonstrates informed insight into their privileged social location and becomes motivated to engage in Agent Advocacy by advocating among or between agents. For example, this might manifest as an effort to create policy changes to challenge the systems of power and privilege. This experience also involves awareness that the burden of social justice action should not fall on oppressed groups but is the responsibility of persons with social privilege. Agent Advocacy is distinct from Empty Advocacy insofar as it has a catalytic effect on other agents, organizations, and policies within systems of power and oppression. This new awareness can be both empowering and a relief; the individual is emboldened to engage in advocacy with other agents and relieved in feeling they have finally found an “answer” or “way out” of the discomfort of Stage Three.

In addition to recognizing the responsibility to engage in Agent Advocacy, this stage accepts that being an agent of change requires risk. Agent Advocacy can be experienced as a dangerous or threatening action because it requires a person to risk their privileged position and voluntarily differentiate themselves from the prevailing social order. This type of advocacy requires Agent Compassion for self, for other agents, and for those for whom the individual is advocating. A participant described their Agent Compassion as they stated:

I should not expect people to always line up with me and it does not mean they are behind or they are, you know, less intelligent than me. It just means that they have had a different life and have a different journey with all of this [privilege awareness].

The data indicate that Reconciliation is not an end goal or final destination of social privilege awareness. The experience of Reconciliation can be fleeting, and individuals might quickly cycle through other stages after experiencing Reconciliation. However, peer debriefing suggests once a person experiences Reconciliation, they become more informed of the process of social privilege awareness and might be able to move through each stage with more ease and less discomfort in the future.

## Discussion

This MIAPSI emphasizes the importance of flipping the coin of oppression to focus on socially-conferred privilege, and offers new insights for doing so. Mirroring previous findings in psychological literature, the interviews we conducted immediately reflected participants’ lack of understanding of social privilege. Although some participants articulated a definition of social privilege that might have qualified for “nuanced understanding” of Spanierman & Smith (2017, p. 609), many participants were eager to hear our own definition of privilege when we offered it. In aggregate, participants demonstrated all aspects of definition of privilege of Black and Stone (2005) while implicitly linking it to oppression (King, 1991; Goodman, 2015), though not all participants could easily define privilege. When we provided a definition of social privilege, we often witnessed participants going through each of what would later become the stages of the model. Reflecting again on the interviews after construction of the model, then applying this model to our interactions with colleagues and students, helped to clarify what this model adds to the current literature within psychology.

First, and perhaps most obviously, this model offers additional focus on social privilege as a general construct. This is a distinct departure from other models which describe the development of a specific target identity domain (e.g., race, gender, and citizenship). Lewis (2004) points out that attempting to describe a single identity domain risks essentializing that group despite clear evidence for heterogeneity of self-consciousness among members of that identity domain. Thus, the benefit of articulating a developmental process of social privilege more broadly is increased accessibility and application to any identity domain with less risk of essentializing a group or of pinning an individual to a distinct process when they might resonate with a more diffuse process. Case and Cole (2013) directly addressed the struggle with resistance to privilege that many educators face by encouraging attention to alternative privileged identity domains to facilitate reflection and progress.

Second, this model acknowledges the effortful intrapsychic process that accompanies linking oppression to social privilege. Although we encouraged participants to speak about their agent vs. target ranks, not all participants could explicitly describe the benefits of their privileged positionality. Individuals who did often reflected on the shame and frustration they felt about their positionality. Sustaining awareness of social privilege requires effort that is worthy of recognition and acknowledgement. The concept of social privilege represents an alternative vision of society in which various myths—the instruments of dysconsciousness—are discredited and replaced with structural and material inequities that uphold privileged ranks. Acknowledgement of this alternative vision requires critical consciousness of what has previously been essential to comfortable dysconsciousness: the identity narrative that asserts we are who we are by our own volition. In order to reconcile the dissonance that results from the incongruence of social privilege with the identity narrative, an individual must confront this threatening reality by protecting their identity narrative through avoidance (dilution), externalization (defense), and grandstanding (empty advocacy), or by changing their identity narrative to accommodate the new perspective (reconciliation).

Third, this model normalizes the crises that occur as awareness of social privilege develops and de-centers the concept of “self” as the primary issue of oppression. Discomfort often arises when a wrong has been named. In the case of becoming aware of social privilege, the wrong is not the privilege of the individual’s “self,” but the ideologies that have perpetuated the focus on “self” and obscured the structures that produce material inequity. As Helms (2017, p. 719) wrote, “[n]aming a wrong means that one has the possibility of ending it.” To neglect or problematize the dissonance inherent to these crises would undermine the importance of these crises to the developmental process.

Fourth, this model offers a developmental map to bridge the gap between unaware privilege and sustained, sensitive advocacy. All of the participants in this study were somewhere between dysconsciousness of privilege and enacting perpetual self-reflection on their privilege, which are the dichotomous points that have been emphasized in the multicultural competence literature. Although a varied topography of development became clear during analysis of the interviews, no participant recognized the developmental nature of their own process and, therefore, some participants expressed a sense of isolation and stuckness. That individuals find privilege threatening was clearly evident among these participants, and a “woke/not woke” dichotomy hampered individuals’ effectiveness in navigating this threat. This model offers directional markers to decrease isolation and increase self-efficacy.

Finally, this model describes factors conducive to developing awareness of social privilege and reconciling the threat it poses. Just as Bennett (1986) describes essential tasks for an individual to move from one stage to the next, this developmental model of social privilege awareness identifies interpersonal, intrapersonal, and environmental factors that facilitated participants’ effective response to awareness of social privilege. Participants who had self-compassion, curiosity, early and continued exposure to a sociohistorical framework of privilege, and a stability of identity were more often able to effectively reconcile social privilege with their identity narratives; this knowledge has immense implications for what clinical psychology can offer to students, trainees, colleagues, and clients developing an awareness of their social privilege.

Helms (1984) stressed psychology’s history of placing undue emphasis on the experiences of oppressed groups, which obscures the role of privileged groups in maintaining institutional oppression. Following Helms’ call to action, theories of racial identity development for White persons have been established; however, theories specific to persons with social privilege and power in American society that help psychologists to understand “why some individuals transform and others do not” (Sue, 2017, p. 712) have not yet been established. This model provides a developmental explanation.

### Model comparison

Returning to existing developmental models of social identities, both Helms’ WRID model and Hardiman and Jackson’s (1997) five-stage social identity development model (SIDM) emerge with clear parallels to the current study’s model. Watt (2007) describes common reactions to discussions about privileged identity in a third model, the Privileged Identity Exploration (PIE) model, which we will also review here.

Although the WRID model measures the construct of White racial identity, the model’s six stages share many similarities with the current study’s model. For example, WRID’s first stage is *contact*, which occurs “[a]s soon as one encounters the idea or the actuality of Black people,” and this stage can occur ether “vicariously or directly” (Helms, 1990, pp. 54–55). Contact is akin to DSPIM’s first stage, Critical Exposure, as both speak to an initial encounter with the Other that can take place either through direct contact or by becoming informed of the Other. Helms (1990, p. 58) suggested individuals might remain in contact depending whether they had a vicarious or direct exposure, as well as when “socialization experiences penetrate the White person’s identity system.” Similarly, Bergkamp et al. (2022) suggested individuals might have multiple exposures to difference, but do not enter critical exposure until they have an experience that punctures their dysconsciousness.

The second stage of the WRID is *disintegration*, an “[a]wareness of the social implications of race on a personal level” (Helms, 1990, p. 68) that often presents a conflicted experience of Whiteness. This stage mirrors MIAPSI’s second stage, Identity Threat, as both authors describe experiencing dilemmas about oneself and the world. Helms suggested, “During this stage, the person may feel caught between White and Black culture, oppression, and humanity” (1990, p. 68). There is a pull to recognize oppression and a resistance to this recognition because it inherently involves an acknowledgment of how one is a benefactor of oppression. Identity Threat entails dissonance and confusion and describes Helms’ conceptualization of moral dilemmas as a “paradox of power.”

The third stage of WRID, reintegration, occurs when a White person’s feelings of guilt and anxiety about their Whiteness morphs into fear and anger toward Black people. This stage is similar to aspects of MIAPSI’s Identity Protection stage, specifically defense. During Defense, due to the threat of their own social privilege, agents’ dissonance can manifest as fear or anger toward targets. Thus, agents might make racist comments, engage in sexist behaviors, or unconsciously act out their classism or xenophobia by suggesting homeless people are “lazy” or immigrants are “taking jobs away.” Likewise, Helms suggested during reintegration, “Anger [is] covertly or overtly expressed as well as [a] projection of one’s feelings” (1990, p. 68).

Pseudo-independence is the fourth stage of WRID and occurs when a White person rejects notions of White superiority and Black inferiority. In this stage, Helms stated, “The person has an intellectual understanding of Black culture and the unfair benefits of growing up White in the United States” (1990, p. 68). White people, therefore, begin to try and redefine their Whiteness, but not necessarily in a positive way. Pseudo-independence resembles aspects of Bergkamp and colleagues’ third stage, Identity Protection, and specifically the soothing strategy of empty advocacy. The fifth stage of WRID, Immersion-Emersion, reflects a White person’s relocation of the problem of racism from the “other” to the self. This turn marks the beginning of an emotional experience that builds on cognitive experiences of White identity developed during the Pseudo-Independence stage. This process is related to the current study’s finding that both cognitive and affective dissonance as well as comparative exposure were required for a change in identity narrative to occur.

Finally, the sixth stage of WRID, Autonomy, takes place when a White person is able to achieve a “bicultural or racially transcendent worldview” (Helms, 1990, p. 68). This last stage consists of the White person internalizing a positive anti-racist identity and is conceptualized as an “ongoing process” (Helms, 1990, p. 66). The final stage of MIAPSI, Reconciliation, reflects autonomy in that they also describe an integration of a new, social-justice oriented identity and, therefore, a change in self.

While not as widely recognized as WRID of Helms (1984), Hardiman and Jackson’s (1997) five-stage social identity development model (SIDM) specifically explores the construct of social privilege identity development. Although SIDM does not reflect the same developmental process, Hardiman and Jackson’s model shares similar conceptual properties with MIAPSI. For example, both MIAPSI and SIDM capture conscious and unconscious experiences and expressions of privilege.

The third stage of SIDM, resistance, is also reflective of different aspects of MIAPSI stages. Hardiman and Jackson’s resistance is characterized by an opposition to the oppressive status quo. Individuals begin to question oppressive ideology and acknowledge and understand their own participation in the system. In describing Hardiman and Jackson’s model, Goodman (2011) notes resistance is characterized by a questioning of one’s identity and sense of self in a newly acknowledged unjust world, a phenomenon that occurs in MIAPSI’s Identity Threat.

The fourth stage of SIDM, redefinition, involves the redefinition of an individual’s identity as well as the dominant group of which they belong (Hardiman and Jackson, 1997). This stage ultimately results in a more complex understanding of self as well as the system of oppression, which is reflective of MIAPSI’s Reconciliation stage, specifically integration. Finally, the fifth stage of Hardiman and Jackson’s model is internalization, which occurs “Once people become comfortable with their new sense of identity” and “people at this stage need peers or organizations where there are people who share their perspective and can affirm this sense of identity” (Goodman, 2011, p. 47). Internalization also mirrors MIAPSI’s Reconciliation stage, especially agent-to-agent advocacy.

Despite similarities, there are also important dissimilarities between MIAPSI and WRID, and MIAPSI and SIDM. In comparison to WRID, first, the third and fourth stages of model of Helms (1990) are represented in aspects of the third stage of MIAPSI. There is, therefore, dissimilarity in the trajectory of development. Second, sixth stage of Helms (1990) differs from MIAPSI’s Reconciliation in that agents cannot have a transcendent experience. Instead, MIAPSI’s agents realize they are stuck within a system that they want to begin to change but from which they cannot escape. Third, whereas Helms (1990) suggested individuals remain in autonomy and continue to experience growth in this stage, MIAPSI suggests agents cycle through the beginning stages of their model, and can reach reconciliation for one or more social identity domains multiple times. Finally, the two models explore different constructs. While WRID describes White identity development, MIAPSI describes social privilege integration, which inherently incorporates identity development. Further, MIAPSI assumes that Whiteness is only one of 10 types of social identity privilege.

In comparison to SIDM, MIAPSI’s developmental process is holarchical whereas SIDM is conceptualized as sequential (Goodman, 2011). A significant distinction is that SIDM’s stages suggest individuals only begin to actively or passively participate in an unjust system once they become aware of it. In contrast, MIAPSI suggests agents actively and passively participate in systems of social privilege whether or not they are conscious of social privilege. In addition, SIDM suggests individuals can begin actively resisting discriminatory attitudes and working to change oppressive policies in the third stage before individuals begin redefinition and integrate social justice principles to their new identities. The MIAPSI, however, indicates that social justice action is illustrative of their final stage, Reconciliation.

Watt (2007) developed the PIE model for anticipating common defensive behaviors that obstruct productive dialogue about diversity, privilege, and social justice, especially on campuses of higher education. As a facilitator of learning, Watt reflects on the utility of this model for building both self-compassion and compassion for learners in the face of defensive behaviors. We believe MIAPSI provides a framework of the process underlying such commonly seen defensive behaviors as well as some evidence for self-managing behaviors during Reconciliation, which not only facilitate compassion for self and others but also provide a foundation for building on behavioral strengths toward social justice action.

Three categories of PIE encompass eight defense modes that arise when exploring the concept of privileged identity. Although Watt does not present this as a developmental model, the processional stages of MIAPSI connect in some ways to the PIE categories which range from initial presentation of new knowledge of privilege to socially just action. The PIE categories of Recognizing, Contemplating, and Addressing similarly describe an evolving orientation to new awareness of privilege. The defense modes within each of these categories suggest a deepening awareness of the implications of privileged identity while ultimately keeping the implications for one’s identity narrative at bay. Although fewer, the strategies described in Stage Three of MIAPSI (Identity Protection) relate to many of the defense modes of PIE. Dilution (MIAPSI) seems to encompass Deflection, Rationalization, Intellectualization, and Minimization (PIE), while Denial neatly maps with Denial and Empty Advocacy (MIAPSI) aligns with Benevolence (PIE). Although we have seen False Envy and Principium in action, these types of behaviors did not emerge from the data as coherent strategies during our analysis.

## Limitations

Purposeful and convenience sampling were the primary recruitment strategies in an attempt to both minimize self-selection and achieve a broader range of experiences with privileged social identity. Despite our desire to minimize self-selection among participants, there was likely an inherent bias in the participants due to their varying relationships with the authors situated in higher education and the field of psychology. Participants included relatively close friends or acquaintances of the original seven members of the research team and did not exclude classmates or students in other health service fields. The researchers’ background and graduate-level education in clinical psychology increases the likelihood of limiting the range of participant experiences, thoughts, feelings, and ultimately data collection and theme generation. However, given the often provocative or even contentious nature of conversations about social privilege, the research team determined to conduct interviews with individuals with whom they already had friendly or familiar relationships; this helped to maintain emotional safety for both the interviewers and the interviewees.

Although all participants reported a privileged social identity and some experience of becoming aware of its implications, the status of more than half of the individuals as students in a social justice graduate program might afford them greater self-awareness or dedicated time for self-reflection within the graduate curriculum. Some readers might view these participants’ unique position as diminishing the applicability of MIAPSI to individuals not in a psychology training capacity. However, by the principles of fit and work, MIAPSI is also grounded in the reported experiences of individuals who do not have a student or trainee status, thus maintaining its applicability to individuals who hold a privileged social identity regardless of such status.

The sample of 18 participants ranged in age from mid-20s to early 60s; 10 participants were psychology graduate students; 13 participants were women; three were men; and 16 participants identified as racially White. Given participant demographics, the sample is considered to be a primarily WEIRD (White, Educated, Industrialized, Rich, and Democratic; Heinrich et al., 2010) sample. However, given that the current study aimed to explore the development of social privilege awareness, the researchers intentionally sought participants with primarily privileged social locations; the demographics of participants are thus seen as a strength. Although there might be similarities of experience between the American participants in our sample and European individuals with privileged social identities, there are likely more differences based in diverging colonial histories which bear comparative study in the future.

## Future directions for research

This grounded theory is the first developmental model that addresses general social privilege awareness across the identity domains. It shares many aspects from other racial identity and privilege awareness models, but highlights the journey an individual makes from initial awareness to personal integration. Here we offer ideas for further research to elucidate this preliminary model, moving stage by stage.

First, the concepts of dysconsciousness and hegemonic socialization sustain a shared state of unawareness for participants. We hypothesize that these two concepts, among others, serve to maintain systems of oppression. As such, they are the main barriers to privilege awareness throughout the process. Further research into the instruments of dysconsciousness and the nature of hegemonic socialization would assist the goal of dismantling oppression at the source. This includes further conceptual refinement, as well as possible measurement and correlations with variables such as life history and social influences.

Regarding Stage 1, Exposure, the main question appears to be what factors are necessary to reach critical consciousness. Why do some individuals require just a few cognitive or comparative exposures in order to spur critical awareness while others may be inundated yet remain oblivious to their privilege? And what is the role of volition in this initial stage, as some individuals actively seek alternative perspectives whereas others are required to attend classes or training that may elicit privilege awareness? We noticed some relationship between critical exposure and developmental life stages, in which young adults appeared most likely to be open to consideration of privilege awareness. We hypothesized that this may be due to a lack of detrimental social costs as there were minimal dependent relationships during this life stage (such as parents or intimate partners). Research on exposure could include classifying types of exposure, such as cognitive and comparative, and exploring the duration, depth, and emotional salience of the exposure. Considering the holarchical nature of MIAPSI, further research across the lifespan of individuals with privileged social identities and across the career-span among practitioners in psychology could illuminate how exposures reach that critical point. Finding the most conducive exposure could prompt initial awareness more effectively. In addition, participants seemed to be able to remember their critical exposure, somewhat like an origin story, which can make research on this stage easier.

Identity Threat, the second stage, involves the psychological concept of dissonance. Dissonance and identity issues have a deep literature within psychology, as the heart of the therapeutic endeavor is to explore and modify habits of self. Both cognitive and affective dissonance can serve as a powerful motivation for change or keep individuals stuck in habitual tendencies. The realm of social privilege awareness was no different. Also, we know that change is more likely when there is a supportive social structure in place. The intensity and duration of dissonance, as well as the type (cognitive and affective) could be studied and compared to the level of social support an individual possesses. One of our hypotheses is that more social support for privilege awareness would lessen the intensity and duration of this stage, and vice versa.

Identity Protection, the third stage, was the most elusive for participants to describe. Strategies of protection (Defense, Dilution, and Empty Advocacy) were more actively demonstrated than articulated. Yet, after these categories were defined, they appeared fairly obvious in the data. Further, this stage appeared to be prone to the most stuckness, in congruence with the general purpose of the stage—to avoid true integration into one’s identity. This was the stage in which the hegemonic views that were unconsciously socialized began to find explicit expression in cognitive, emotional, and behavioral ways. Of note, defense can appear aggressive and attacking in nature, yet we interpreted this as a protection strategy versus outright intentional violence. This does not negate the harmful nature of the behavior or the need for accountability. Additionally, though it appeared that these strategies were utilized in a somewhat consecutive order (Defense-Dilution-Empty Advocacy), it is unclear if that was specific to our dataset or indicative of a general pattern. Future research can further clarify these strategies, in what contexts they are most likely to occur, and the level of intentionality when utilized.

A hallmark of this model is the conducive factors of interpersonal and intrapersonal safety, and cognitive scaffolding that supports someone taking risks in modifying their identity narrative in light of social privilege. The data revealed the power of agent-to-agent support in order to move from Identity Protection to Reconciliation. Cognitive Scaffolding was essential to provide a framework for individuals to understand the disruptive impacts of privilege awareness. Interpersonal Safety, in the form of strong relationships with others that could withstand identity change, allowed individuals to brave the idea of identity narrative change. Intrapersonal Safety, having an intact but pliable sense of self that can withstand narrative revisions, was essential in order to let go of protection strategies and seek a more congruent personal story.

Although this study identified these conducive factors, further research could assist in clarifying the concepts of Interpersonal and Intrapersonal Safety and Cognitive Scaffolding and their interactions. Specifically, is Intrapersonal Safety dependent on social support and cognitive frameworks? Is there an order to these factors, such as Interpersonal Safety first, then Cognitive Scaffolding, then Intrapersonal Safety? Is it possible to move from Protection to Reconciliation without these conducive factors, and how does this process occur?

The conducive factors also appear to relate to the idea of “brave spaces,” where agents unburden targets to provide a pathway, and instead unpack issues of social privilege with others that hold the same agent ranks. How do these groups provide and cultivate the conducive factors? Further research could assist in honing these concepts and tailoring specific interventions to cultivate the transition to integration of privilege awareness. Investigation of any connections between relationships built in therapy and these conducive factors could increase the specificity of MIAPSI for practitioners in psychology aiming to follow the American Psychological Association (2017) multicultural guidelines for both self-study and clinical application.

The ongoing stage of Reconciliation involves acceptance, empathy for the agent developmental process, and advocacy for agent change. Acceptance is ultimately about standing still and calm in the light of social privilege awareness and allowing it to change the way we perceive ourselves and the world. This includes changes to our personal narrative, our relationships, and our attributions. Out of this process, the individual can also empathize with other agents moving through the process. They can relate to the shock of exposure, the dissonance of threat, and the misinformed moves to protect. From this point, individuals are motivated toward systemic change through agent advocacy, meaning encouraging other agents in their own identity revision.

Future research regarding Reconciliation can help identify the behavioral markers of this stage, as well as the interaction between acceptance, empathy, and agent advocacy. As we hypothesize, acceptance is the first and essential element of this stage, with empathy and advocacy subsequently resulting. We further assert that once reconciliation is achieved in one agent rank (race, gender, SES, etc.), it is more likely to occur for others, as the process is familiar and the support from the conducive factors have been utilized.

## Implications for diversity education

It has become increasingly important to identify whether individuals have an awareness of social privilege, especially as a lack of social privilege awareness might result in harming vulnerable or marginalized persons, committing overt and covert acts of interpersonal aggression and discrimination, and perpetuating oppressive systemic patterns. We set out to understand a broad process that would help us as practitioners and learners in psychology understand this awareness process. Although we see many applications and future directions of this study, we see diversity education as the most proximal starting point for application of MIAPSI. For many of the student participants in our study, their opportunities to critically engage with the concept of social privilege were limited prior to university or even graduate education. In addition to aiding the practice and research of psychology, developmental social privilege awareness models can also benefit the education and training of psychologists.

Although there are challenges in defining social justice (Thrift and Sugarman, 2018), scholars have described social justice within psychology to involve advocacy (Motulsky et al., 2014) and “recognition of the impact of unearned privilege and discriminatory oppression on clients’ mental health” (Singh et al., 2010, p. 767). Scholars have also advocated for psychologists to become change agents, which involves “scholarship and professional action designed to change societal values, structures, policies, and practices, such that disadvantaged or marginalized groups gain increased access to tools of self-determination” (Goodman et al., 2004, p. 793).

Despite psychologists’ recent call to action, there is a dearth of literature offering approaches, standards, and outcomes for implementing doctoral-level social justice pedagogy in psychology curricula. A significant portion of the existing literature reveals that counseling, educational, community, critical, and liberation psychologists (Goodman et al., 2004), as well as masters-levels programs, have engaged more in social justice work. Further, much of the literature focuses on social justice philosophies, definitions, and competencies (Ali et al., 2008; Singh et al., 2010; Motulsky et al., 2014); there has been little effort put forth in outlining practical implementations of social justice, in not only multicultural competence but across all doctoral-level psychology curricula.

Motulsky et al. state that “Although more programs integrate multicultural content across the curriculum, it is unusual for social justice issues to be incorporated into the majority of the coursework” (p. 1062). While authors such as Case and Cole (2013) have helped to establish the importance of social privilege within social justice pedagogy, Singh et al. (2010) found that among 66 doctoral-level psychology trainees, 85% had not taken a course with social justice content and reported disparities in their definition of social justice. However, Singh et al. also found that many of the participants strived to incorporate social justice into their practice of psychology and sought training outside of their academic institutions. Singh et al.’s study signifies a clear need and appetite for social justice pedagogy among psychology trainees. Further, Bartoli et al. (2015) assert that “Facilitating multicultural competence has become central to ethical clinical counseling training, with its responsibility resting on training programs and clinical supervisors” (p. 247). Given the APA’s social justice aspirations outlined in their 2017 Multicultural Guidelines, it has become critical for doctoral-level psychology programs to incorporate social justice pedagogy into their education and training of future psychologists.

Vera and Speight (2003) argue that social justice can be incorporated into psychology programs by training the next generation of psychologists as change agents. With the inclusion of social justice pedagogy, a model such as this could have significant implications on educating and training incoming psychologists. This could help identify trainees’ stages of development and inform the types of education and support they might need. Further, the model can help to track the progress of the trainee’s development. Finally, the model can help to assess the efficacy of multicultural and social justice psychology courses by gathering data before and after the courses.

## Summary

Clinical psychologists hold a unique position as agents of change across nearly all ecological levels (Bronfenbrenner, 1979; American Psychological Association, 2017), particularly for social justice. Yet, in pursuit of understanding social privilege as a major contributor to oppression and inequity, psychologists are easily stymied by minimal research in this area. A unified definition of social privilege (e.g., Black and Stone, 2005) and empirical research in the process of becoming aware of a privileged social identity (e.g., Helms, 1990) are necessary tools for clinicians, educators, and consultants within the field of psychology. The holarchical and developmental Model of Integrating Awareness of a Privileged Social Identity (MIAPSI) presented here adds to the growing body of empirical research and literature to support psychologists and psychologists-in-training coming to terms with both their own and their clients’ and students’ privileged social identities on a broad scale. Practitioners within the field of psychology can benefit from naming their Critical Exposure, their experience with Identity Threat and efforts at Identity Protection, and illuminating the Conducive Factors they experienced or might look for in support of Reconciliation. On the journey from agent to ally (Spanierman and Smith, 2017), MIAPSI provides a framework for understanding the process of integrating awareness which can be applied not only to self but to others in pursuit of social justice.

## Data availability statement

The raw data supporting the conclusions of this article will be made available by the authors, without undue reservation.

## Ethics statement

The studies involving human participants were reviewed and approved by Antioch University Seattle. The patients/participants provided their written informed consent to participate in this study.

## Author contributions

JB served as the primary investigator for this project, as well as study conceptualization. LO and AM coordinated the data collection. JB, LO, and AM worked equally on the writing of the manuscript. All authors contributed to the article and approved the submitted version.

## Conflict of interest

The authors declare that the research was conducted in the absence of any commercial or financial relationships that could be construed as a potential conflict of interest.

## Publisher’s note

All claims expressed in this article are solely those of the authors and do not necessarily represent those of their affiliated organizations, or those of the publisher, the editors and the reviewers. Any product that may be evaluated in this article, or claim that may be made by its manufacturer, is not guaranteed or endorsed by the publisher.
